# Unveiling nitrogen preferences in *indica* rice: a classification study of cultivars in South China

**DOI:** 10.3389/fpls.2025.1568383

**Published:** 2025-04-28

**Authors:** Chu-sheng Lu, Jia-jun Lai, Xian-ting Fan, Kai-ming Liang, Yuan-hong Yin, Qun-huan Ye, Hong Shen, You-qiang Fu

**Affiliations:** ^1^ Rice Research Institute, Guangdong Academy of Agricultural Sciences, Guangzhou, China; ^2^ Guangdong Key Laboratory of Science and Technology in Rice, Guangzhou, China; ^3^ Key Laboratory of Genetics and Breeding of High Quality Rice in Southern China (Co-construction by Ministry and Province), Guangzhou, China; ^4^ South China Agricultural University, Guangzhou, China

**Keywords:** ammonium and nitrate preferences, nitrogen treatments, ammonium-nitrate mixed nutrition, classification of indica cultivars, biomass

## Abstract

**Introduction:**

Do *indica* rice cultivars prefer ammonium or nitrate? Understanding this preference is key to optimizing nitrogen use efficiency in rice production. Ammonium and nitrate are crucial for plant nitrogen nutrition, as rice cultivars exhibit varying preferences. However, few studies have classified ammonium and nitrate preferences within *indica* cultivars.

**Methods:**

For the first time, this study classifies *indica* rice cultivars based on their ammonium and nitrate preferences, revealing significant differences in biomass production under various nitrogen treatments. This study investigated the effects of ammonium-only nutrition (100:0), ammonium-nitrate mixed nutrition (75:25), and nitrate-only nutrition (0:100) on the maximum root length, shoot length, SPAD value, and biomass of 24 widely cultivated *indica* cultivars in South China.

**Result:**

Compared to ammonium-only nutrition, a mixed ammonium-nitrate treatment significantly boosted root and shoot growth, while nitrate-only nutrition led to a decline in chlorophyll content. Compared with the 100:0 treatment, the maximum root length, shoot length, root dry weight, shoot dry weight, and total dry weight in the 75:25 treatment significantly increased by 29.85%, 4.11%, 7.65%, 1.71% and 3.03% (p < 0.01), respectively; and the SPAD value in the 0:100 treatment significantly decreased by 4.22% (*p* < 0.01).

**Discussion:**

These results demonstrate distinct responses of rice cultivars to different nitrogen treatments. Through correlation, principal component, and cluster analyses, the rice cultivars were categorized into three types: ammonium-preferring type (APT), ammonium- and nitrate-preferring type (ANPT), and nitrate-preferring type (NPT). The APT, ANPT, and NPT showed the highest biomass in the 100:0, 75:25, and 0:100 treatments, respectively, with the biomass in the ANPT significantly exceeding that of the APT (*p* < 0.01). These insights provide a foundation for breeding high-yield *indica* rice, optimizing nitrogen fertilizer strategies, and improving nitrogen use efficiency in sustainable agriculture.

## 1 Introduction

Nitrogen is an essential macronutrient for plant growth and plays a crucial role in several metabolic functions (Wang et al., 2019). It is integral to the synthesis of amino acids and proteins, the formation of nucleic acids, and the production of chlorophyll (Torre and Ávila, 2021). Nitrogen is also a key component of enzymes and cofactors and is involved in vital processes such as energy transfer and ATP formation (The et al., 2020). Thus, nitrogen significantly affects plant growth, yield, and quality, and these parameters are closely related to the forms in which nitrogen is available to plants (Li J. H. et al., 2022).

In nature, the main inorganic nitrogen forms available for plant utilization are ammonium and nitrate, with their relative dominance being influenced by the soil environment. Nitrate primarily exists in aerobic or alkaline soils, whereas ammonium predominates in anaerobic or acidic soils (Zhang et al., 2019). Consequently, soil conditions play a crucial role in shaping the ammonium and nitrate preferences of crops (Xiao et al., 2023). Dryland crops typically prefer nitrate, whereas aquatic crops favor ammonium. Even under flooded conditions, rice, a species that typically prefers ammonium (Li et al., 2013), can induce nitrification in the rhizosphere through the oxygen released from its roots (Dai et al., 2022). As a result, nitrate uptake can account for up to 40% of total nitrogen uptake (Li and Li, 2016). In recent years, the promotion and application of technologies such as alternate wetting and drying irrigation and aerobic cultivation have led to an increase in the conversion of ammonium to nitrate in soil (Fu Y. Q. et al., 2021; Xu et al., 2023). Consequently, in actual production systems, rice consistently experiences the conditions of ammonium-nitrate mixed nutrition (ANMN).

Numerous studies have demonstrated that a suitable ratio of ANMN stimulates crop growth and development, enhances yield and quality, and improves crop resistance (Liu et al., 2017; Zhan et al., 2023). The appropriate ratio of ANMN has been found to improve the growth, yield, and quality of various dryland crops such as *Panax notoginseng* (Li Q. Q. et al. 2022), maize (Wang et al., 2023), *Brassica napus* (Li et al., 2021), and tobacco (Chen et al., 2023). Moreover, a suitable ratio of ANMN can significantly improve the rhizosphere microenvironment of rice, promote root growth, and increase photosynthesis and stress resistance, thereby increasing biomass and yield (Xu et al., 2020; Jiang et al., 2021). Our previous research demonstrated that compared with those of ammonium-only nutrition (100:0) and other ANMN (50:50 and 25:75), the 75:25 treatment of ANMN was more effective at promoting the growth and nutrient absorption of rice seedlings (Fu et al., 2023). Recently, a meta-analysis by Chen et al. (2024) reviewed the literature from the past 40 years and revealed that, compared with the 100:0 and 50:50 treatments, the 75:25 treatment notably promoted rice growth and development, enhanced photosynthetic efficiency and enzyme activity, and increased rice dry matter. Thus, ANMN is highly important for crop growth and development. However, different subspecies of the rice may respond variably to different nitrogen nutrition. Zhang et al. (2004a) reported significant differences in the maximum absorption rates of ammonium and nitrate among different rice subspecies. Specifically, the maximum absorption rate of nitrate in *japonica* rice was greater than that of ammonium, whereas *indica* rice exhibited similar maximum absorption rates for both forms of nitrogen. Conversely, Xiao et al. (2023) suggested that *indica* rice prefers nitrate over ammonium. The differences in ammonium and nitrate preferences among rice cultivars may account for these variations in absorption. However, research on this topic remains limited in scope at present.

Li et al. (2013) researched ammonium and nitrate preferences among different crops and revealed that rice prefers for ammonium. Zhang et al. (2004b) analyzed the response of *japonica* cultivars to nitrate and categorized them into three types: highly responsive, moderately responsive, and unresponsive. However, to date, there has been limited research on classifying the ammonium and nitrate preferences among *indica* cultivars. Zhang and Chu (2020) found that *indica* rice usually exhibits higher nitrogen use efficiency than *japonica* rice, mainly due to differences in their ability to utilize nitrate nitrogen. The research by Fu et al. (2024) showed that there are significant differences in nitrogen use efficiency and yield between hybrid and conventional *indica* cultivars. Therefore, this study selected conventional and hybrid *indica* cultivars that were widely cultivated in South China and had differences in nitrogen response to investigate the effects of different nitrogen treatments on the maximum root length, shoot length, SPAD value, and biomass. Correlation analysis, principal component analysis, and cluster analysis were used to classify *indica* cultivars by their ammonium and nitrate preferences, revealing specific morphological indicators of different *indica* cultivar types. These findings provide a theoretical foundation for classifying rice cultivars based on their ammonium and nitrate preferences and offer theoretical guidance for targeted breeding, nitrogen fertilizer optimization, and improved nitrogen use efficiency.

## 2 Materials and methods

### 2.1 Tested cultivars and germination

The tested cultivars included 24 widely cultivated *indica* cultivars in South China, including 13 hybrid *indica* cultivars and 11 conventional *indica* cultivars. The hybrid *indica* cultivars included MeiLiangYou1512 (MLY1512), Guang8You165 (G8Y165), DiYou1512 (DY1512), Guang8You2168 (G8Y2168), Guang8YouJinZhan (G8YJZ), ShenYou9516 (SY9516), TianFengYou3550 (TFY3550), TianYou3301 (TY3301), WuFengYou615 (WFY615), QingXiangYou19Xiang (QXY19X), JinLongYou1512 (JLY1512), JuLiangYou751 (JLY751), and WuYou308 (WY308). The conventional *indica* cultivars included GuangHui128 (GH128), W6827 (W6827), GuangHui751 (GH751), YueHeSiMiao (YHSM), YueXiang430 (YX430), GuiChao2Hao (GC2H), LiXiangZhan (LXZ), WuShanSiMiao (WSSM), YueYaSiMiao (YYSM), NanJingXiangZhan (NJXZ), and HeGuangSiMiao (HGSM).

Seed germination procedures involved selecting plump seeds and soaking them in a 5% sodium hypochlorite solution for 20 minutes, followed by three rinses with pure water. The seeds were then incubated at 30°C in darkness for 36 hours using a light incubator (MGC-450BP). Afterward, they were transferred to Petri dishes containing double-layered moist filter paper for an additional 36 hours of germination. The germinated seeds were selected and transferred to black water culture boxes for different nitrogen treatments.

### 2.2 Experimental design

The experiment was conducted in a glass greenhouse at the Rice Research Institute, Guangdong Academy of Agricultural Sciences, located in Tianhe District, Guangzhou, Guangdong, China. In order to eliminate the influence of environmental factors on nitrogen uptake, automatic temperature and humidity control device was activated during the experiment as follows: the average temperature was 30°C, the average humidity was 60%, and the light conditions were natural sunlight. Three nitrogen treatments were established based on our previous work (Fu et al., 2023): (1) 100:0, 100% ammonium treatment; (2) 75:25, 75% ammonium and 25% nitrate mixed treatment; and (3) 0:100, 100% nitrate treatment. The total nitrogen level for all treatments was standardized to 0.95 mmol·L^–1^. Ammonium was provided by (NH_4_)_2_SO_4_, and nitrate was provided by Ca(NO_3_)_2_·4H_2_O. Following the recommendations of the International Rice Research Institute, all nutrients except nitrogen were added at one-third of the strength of the modified nutrient solution (as described below) to accommodate the growth requirements of the rice seedlings. The pH of the nutrient solution was adjusted to 5.50 ± 0.05 every 12 hours using a 1 mmol·L^–1^ HCl or NaOH solution, and the nutrient solution was replaced every 3 days. After 7 days of treatments, the plants were harvested and the maximum root length, shoot length, SPAD value, and biomass were measured. Each treatment was replicated three times.

The formula of the modified nutrient solution refers to Fu et al. (2023). The major nutrients were as follows (mmol·L^–1^): KH_2_PO_4_, 0.32; K_2_SO_4_, 0.84; CaCl_2_, 1.00; and MgSO_4_·7H_2_O, 1.70. The micronutrients were as follows (μmol·L^–1^): MnCl_2_·4H_2_O, 9.10; H_2_MoO_4_, 0.52; H_3_BO_3_, 18.00; ZnSO_4_·7H_2_O, 0.15; CuSO_4_·5H_2_O, 0.16; and Fe(III)-EDTA, 75.00. To suppress environmental microbial nitrification and prevent the conversion of ammonium to nitrate, 7.00 μmol·L^–1^ dicyandiamide (a nitrification inhibitor) was added to all treatments.

### 2.3 Measurement methods

#### 2.3.1 Measurements of maximum root length (cm) and shoot length (cm)

Eight representative rice plants were randomly selected for each replication, and the maximum root length and shoot length were measured with a ruler.

#### 2.3.2 SPAD value (relative unit) determination

Eight representative rice plants were randomly selected for each replication. The SPAD value was measured via a chlorophyll meter (SPAD-502 Plus). The first fully expanded leaf at the top of each plant was chosen for measurement, and readings were taken at the upper, middle, and lower parts of the leaf, and the average was calculated.

#### 2.3.3 Biomass (mg·plant^–1^) determination

Eight plants were retained for each replication. After the seeds were removed, the roots and shoots were separated and placed into kraft envelopes. They were then inactivated in an oven at 105°C for 15 minutes before being dried at 75°C until a constant weight.

### 2.4 Statistical analysis

In this study, IBM SPSS Statistics 26 was used for analysis of variance (ANOVA) and multiple comparisons. Origin 2021 was used for correlation analysis, principal component analysis, cluster analysis, and graphing.

#### 2.4.1 Correlation analysis

The “Correlation Plot” app in Origin was used for analysis and graphing. The data were normalized and subjected to Pearson correlation analysis with two-tailed tests.

#### 2.4.2 Principal component analysis

The “Principal Component Analysis” app in Origin was used for analysis and graphing. Two principal components were extracted, and the principal component scores were obtained.

Composite score calculation formula:


$$\text{Composite score=}\sum_{\text{k}=1}^{\text{n}}{\text{(Principal component score}}_{\text{k}}{\times \text{Variance contribution rate}}_{\text{k}})$$

#### 2.4.3 Cluster analysis

The “HeatMap Dendrogram” app in Origin was used for analysis and graphing. Composite scores were used as the data, and the average linkage method and Euclidean distance was used for hierarchical clustering.

## 3 Results

### 3.1 Influence of nitrogen treatments on the morphological characteristics of rice seedlings

**Figure 1:**
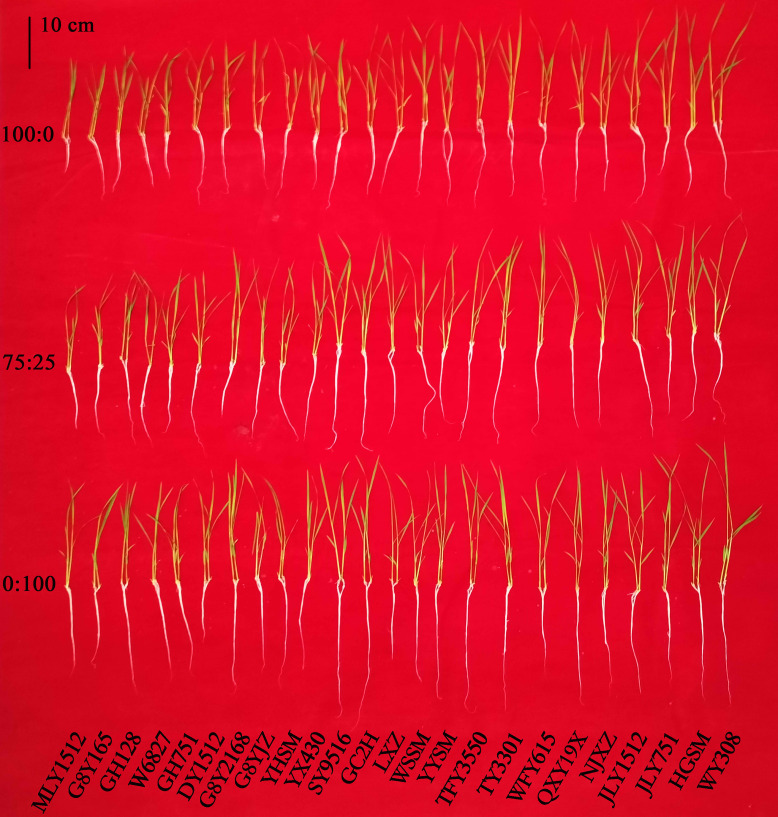
The impact of nitrogen treatments on the growth of various rice cultivars. Each column represents a cultivar, as detailed in Section 2.1. 100:0, 100% ammonium treatment; 75:25, 75% ammonium and 25% nitrate mixed treatment; 0:100, 100% nitrate treatment.

**Figure 1** highlights the varied responses of rice cultivars to nitrogen treatments. ANOVA revealed significant differences among cultivars and nitrogen treatments for the maximum root length, shoot length, SPAD value, root dry weight, shoot dry weight, and total dry weight (*p* < 0.01), with the effect size reaching a medium or higher level (η^2p^ ≥ 0.09). Additionally, interactions between cultivars and nitrogen treatments were observed for all morphological indicators (**Table 1**; **Supplementary Table S1**).

**Table 1:** Effect of nitrogen treatments and cultivars on morphological indicators of rice seedlings.

Factors	Levels	MRL (cm)	SL (cm)	SPAD (relative unit)	RDW (mg·plant^–1^)	SDW (mg·plant^–1^)	TDW (mg·plant^–1^)
Cultivars	MLY1512	13.18 ± 0.96 c	18.04 ± 0.77 f	31.96 ± 0.35 de	2.67 ± 0.15 e	8.34 ± 0.29 f	11.02 ± 0.42 f
	G8Y165	17.28 ± 1.32 bc	17.88 ± 0.89 f	33.10 ± 0.34 cd	3.06 ± 0.06 de	10.77 ± 0.31 de	13.83 ± 0.31 de
	GH128	18.13 ± 1.03 bc	18.91 ± 0.37 ef	36.52 ± 0.50 a	3.36 ± 0.03 cd	10.63 ± 0.30 de	14.00 ± 0.31 de
	W6827	14.95 ± 1.22 c	19.00 ± 0.21 ef	35.01 ± 0.22 bc	3.21 ± 0.18 d	9.58 ± 0.53 ef	12.79 ± 0.68 e
	GH751	14.44 ± 1.19 c	19.89 ± 0.29 de	33.22 ± 0.54 cd	4.48 ± 0.06 ab	12.53 ± 0.41 cd	17.00 ± 0.45 c
	DY1512	19.28 ± 1.08 b	20.10 ± 0.32 de	33.36 ± 0.40 cd	2.53 ± 0.15 e	9.51 ± 0.75 ef	12.04 ± 0.89 ef
	G8Y2168	18.05 ± 1.32 bc	21.86 ± 0.37 cd	31.44 ± 0.45 de	3.18 ± 0.10 d	11.99 ± 0.54 d	15.17 ± 0.64 d
	G8YJZ	15.62 ± 1.17 bc	21.14 ± 0.35 d	28.12 ± 0.61 f	3.14 ± 0.10 d	10.06 ± 0.35 e	13.20 ± 0.44 e
	YHSM	14.45 ± 1.48 c	20.07 ± 0.19 de	32.91 ± 0.28 cd	3.49 ± 0.07 cd	9.89 ± 0.36 e	13.38 ± 0.35 e
	YX430	14.10 ± 1.24 c	19.61 ± 0.25 e	33.67 ± 0.35 c	3.70 ± 0.16 c	10.49 ± 0.41 e	14.19 ± 0.55 de
	SY9516	23.34 ± 1.12 ab	21.92 ± 0.46 cd	34.03 ± 0.48 bc	4.90 ± 0.18 a	16.46 ± 0.22 ab	21.36 ± 0.39 ab
	GC2H	22.98 ± 1.72 ab	23.46 ± 0.63 bc	33.97 ± 0.16 bc	4.83 ± 0.21 a	16.28 ± 0.62 ab	21.11 ± 0.82 ab
	LXZ	18.71 ± 1.23 bc	22.86 ± 0.25 c	31.52 ± 0.21 de	3.76 ± 0.13 bc	12.31 ± 0.37 cd	16.07 ± 0.49 cd
	WSSM	17.63 ± 0.96 bc	21.69 ± 0.20 cd	33.72 ± 0.38 c	4.09 ± 0.07 bc	12.23 ± 0.29 cd	16.32 ± 0.30 cd
	YYSM	18.21 ± 1.27 bc	22.17 ± 0.41 cd	32.76 ± 0.29 cd	3.75 ± 0.14 c	11.17 ± 0.40 de	14.92 ± 0.50 de
	TFY3550	23.73 ± 1.37 a	22.92 ± 0.34 c	32.82 ± 0.23 cd	3.64 ± 0.17 cd	12.71 ± 0.37 cd	16.36 ± 0.54 cd
	TY3301	21.11 ± 1.28 ab	23.32 ± 0.53 bc	30.69 ± 0.52 e	4.17 ± 0.15 bc	15.30 ± 0.33 b	19.47 ± 0.45 b
	WFY615	22.07 ± 1.11 ab	24.51 ± 0.47 b	32.23 ± 0.38 d	3.88 ± 0.21 bc	12.94 ± 0.65 cd	16.82 ± 0.84 cd
	QXY19X	19.24 ± 1.09 b	22.91 ± 0.34 c	31.47 ± 0.38 de	3.54 ± 0.13 cd	11.24 ± 0.56 de	14.78 ± 0.56 de
	NJXZ	17.09 ± 1.38 bc	22.28 ± 0.27 cd	31.94 ± 0.66 de	3.56 ± 0.15 cd	12.95 ± 0.56 cd	16.50 ± 0.70 cd
	JLY1512	20.64 ± 1.17 ab	23.85 ± 0.26 bc	32.48 ± 0.21 cd	3.92 ± 0.19 bc	13.28 ± 0.58 cd	17.20 ± 0.76 c
	JLY751	15.41 ± 1.22 bc	25.46 ± 0.28 ab	35.19 ± 0.70 b	4.61 ± 0.19 ab	15.08 ± 0.17 b	19.69 ± 0.31 b
	HGSM	18.26 ± 1.19 bc	23.71 ± 0.35 bc	35.68 ± 0.26 ab	4.20 ± 0.17 b	13.48 ± 0.54 c	17.68 ± 0.70 c
	WY308	20.05 ± 1.29 ab	26.68 ± 0.37 a	32.19 ± 0.60 d	4.81 ± 0.13 a	17.45 ± 0.32 a	22.26 ± 0.43 a
Nitrogen treatments	100:0	14.07 ± 0.36 C	20.95 ± 0.31 C	33.41 ± 0.22 A	3.53 ± 0.07 C	12.29 ± 0.29 B	15.82 ± 0.35 B
	75:25	18.27 ± 0.34 B	21.81 ± 0.28 B	33.33 ± 0.26 A	3.80 ± 0.10 B	12.50 ± 0.31 A	16.30 ± 0.40 A
	0:100	22.40 ± 0.39 A	22.77 ± 0.28 A	32.00 ± 0.23 B	3.98 ± 0.09 A	12.30 ± 0.35 B	16.28 ± 0.43 A
Multifactorial analysis of variance							
Cultivars (C)		248.60**	248.60**	132.01**	77.24**	319.74**	353.88**
Nitrogen treatments (N)		3724.91**	3724.91**	165.87**	124.79**	310.79**	6.95**
C×N		6.31**	6.31**	7.88**	10.12**	47.79**	48.63**

The data on the cultivars are the average values of three nitrogen treatments, and the data on the nitrogen treatments are the average values of 24 cultivars. The values are presented as the mean ± SE. For multiple comparisons, the least significant difference method and Benjamini-Hochberg correction were used. Different letters in the same column indicate significant differences. Lowercase letters indicate comparisons between the cultivars (α = 0.05); uppercase letters denote comparisons between the treatments (α = 0.01). The cultivars details are provided in Section 2.1. MRL, maximum root length; SL, shoot length; SPAD, SPAD value; RDW, root dry weight; SDW, shoot dry weight; TDW, total dry weight; 100:0, 100% ammonium treatment; 75:25, 75% ammonium and 25% nitrate mixed treatment; 0:100, 100% nitrate treatment; **p < 0.01.

Morphological indicators varied significantly among rice cultivars. The maximum root length ranged from 13.18 to 23.73 cm (80.05% variation), the shoot length from 17.88 to 26.68 cm (49.22% variation), the SPAD value from 28.12 to 36.52 (29.87% variation), the root dry weight from 2.53 to 4.90 mg**·**plant^–1^ (93.68% variation), the shoot dry weight from 8.34 to 17.45 mg**·**plant^–1^ (109.23% variation), and the total dry weight from 11.02 to 22.26 mg**·**plant^–1^ (102.00% variation).

Compared with those in the 100:0 treatment, the maximum root length, shoot length, root dry weight, shoot dry weight, and total dry weight in the 75:25 treatment increased by 29.85%, 4.11%, 7.65%, 1.71%, and 3.03%, respectively. In the 0:100 treatment, the maximum root length, shoot length, root dry weight, and total dry weight increased by 59.20%, 8.69%, 12.75%, and 2.91%, respectively. These differences were statistically significant (*p* < 0.01). Compared with those in the 100:0 and 75:25 treatments, the SPAD value in the 0:100 treatment decreased significantly by 4.22% and 3.99%, respectively (*p* < 0.01).

### 3.2 Correlation analysis

**Figure 2:**
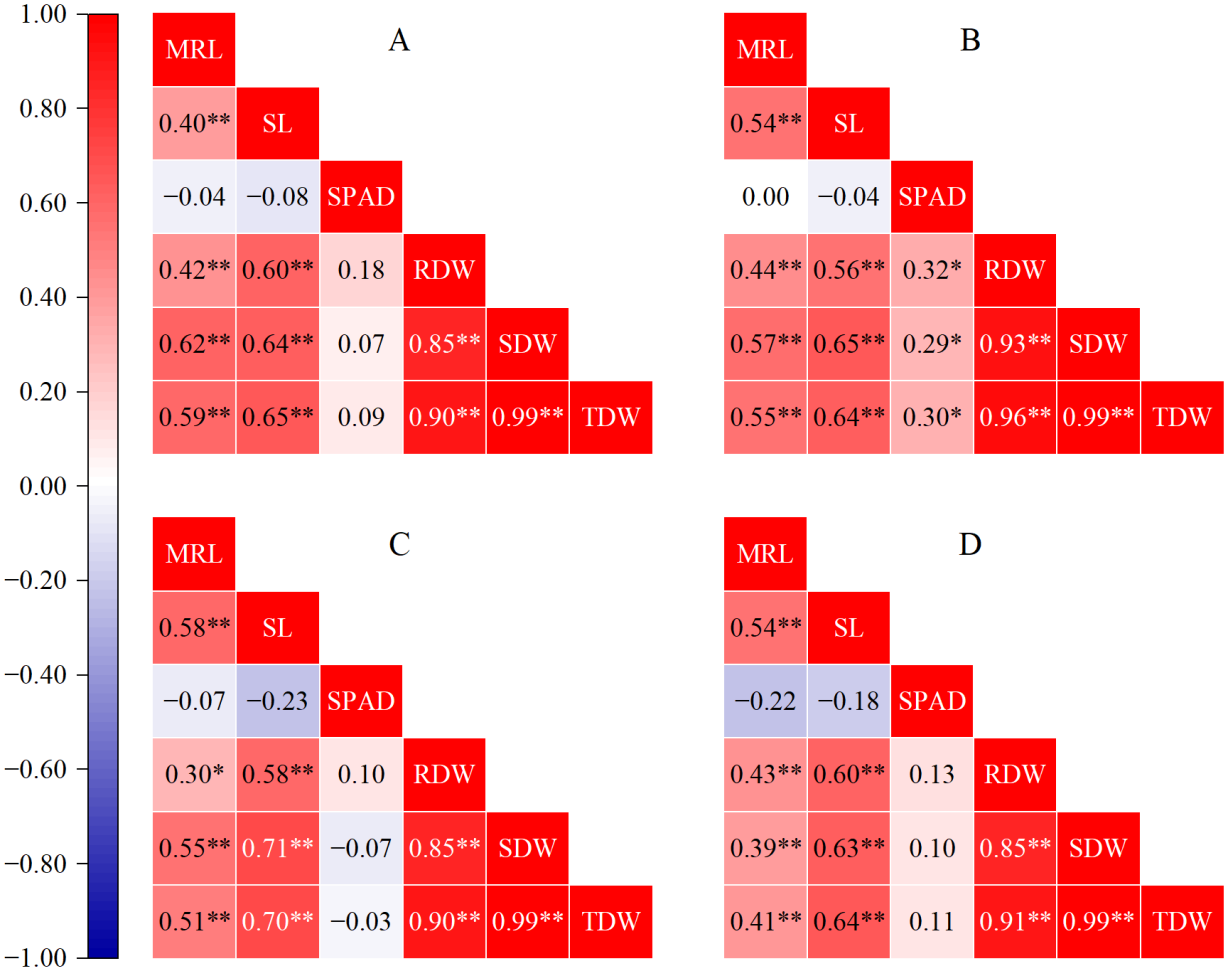
Correlation analysis. The data were normalized and subjected to Pearson correlation analysis with two-tailed tests. The values represent the Pearson correlation coefficient. The Benjamini-Hochberg method was corrected for multiple comparisons. *p < 0.05; **p < 0.01. MRL, maximum root length; SL, shoot length; SPAD, SPAD value; RDW, root dry weight; SDW, shoot dry weight; TDW, total dry weight. **(A)** 100% ammonium treatment, **(B)** 75% ammonium and 25% nitrate mixed treatment, **(C)** 100% nitrate treatment, and **(D)** all treatments.

Correlation analysis of the morphological indicators revealed that there was a highly significant (*p* < 0.01) positive correlation among the maximum root length, shoot length, and biomass in all correlation analyses (**Figures 2A–D**). However, in the 0:100 treatment (**Figure 2C**), the maximum root length was significantly (*p* < 0.05) positively correlated with only the root dry weight. There was no significant (*p* > 0.05) correlation among the maximum root length, shoot length, or biomass in any of the correlation analyses (**Figures 2A–D**). The SPAD value was significantly (*p* < 0.05) positively correlated with the biomass in the 75:25 treatment (**Figure 2B**).

### 3.3 Principal component analysis

**Figure 3:**
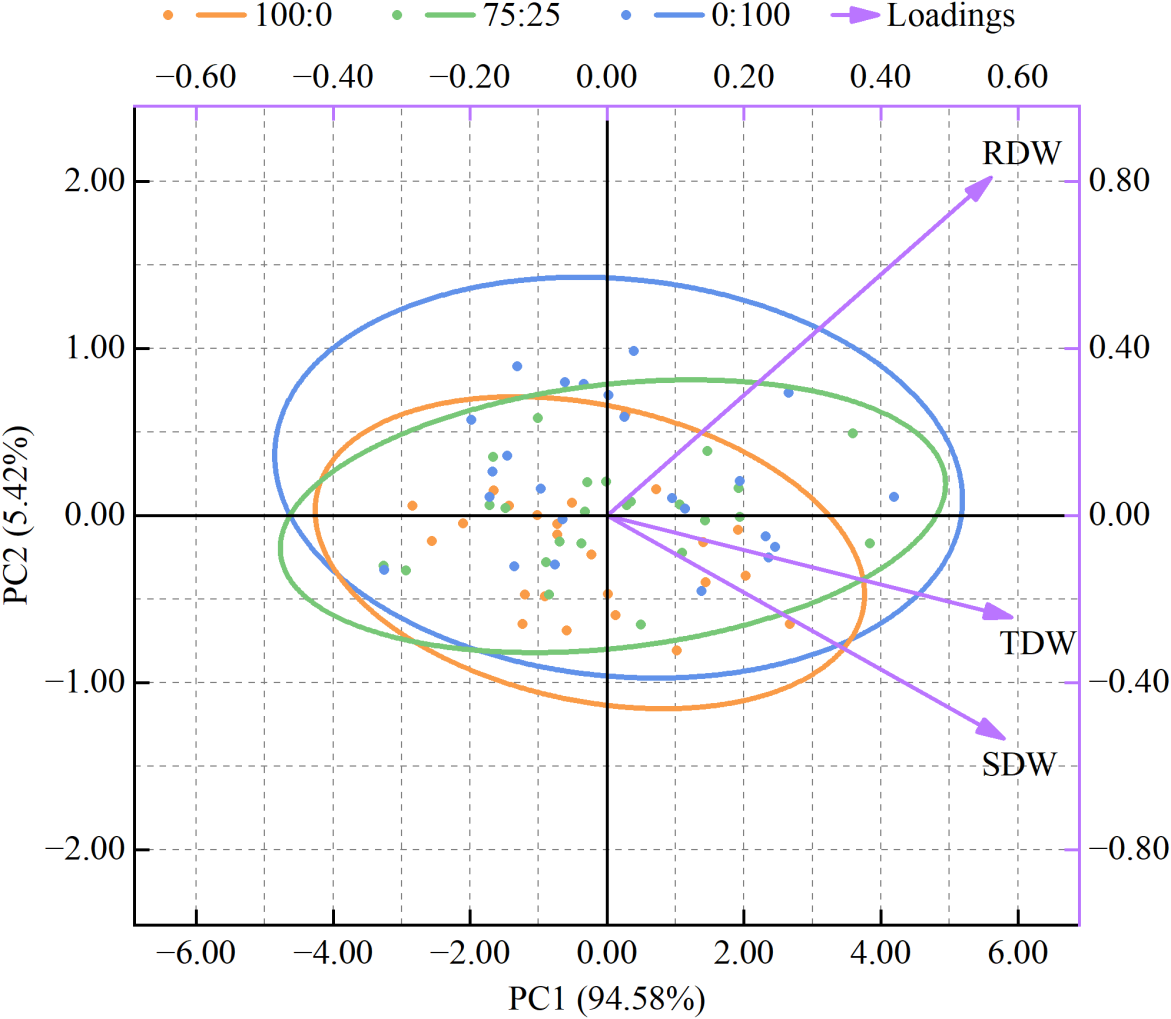
Principal component analysis. 95% confidence interval. The left and bottom axes, and the data points, indicate the principal component scores; the right and top axes and the arrows, represent the loadings. RDW, root dry weight; SDW, shoot dry weight; TDW, total dry weight; 100:0, 100% ammonium treatment; 75:25, 75% ammonium and 25% nitrate mixed treatment; 0:100, 100% nitrate treatment.

To conduct the cluster analysis, dimensionality reduction was performed on the biomass data, resulting in a principal component analysis plot (**Figure 3**). The plot showed that the two principal components could fully explain 100.00% of the data variance. Specifically, the first principal component (PC1) explained 94.58% of the data variance, while the second principal component (PC2) explained 5.42% of the variance. This indicated that PC1 is the primary factor distinguishing sample differences. In the plot, sample points of different colors represented the principal component scores of different treatments. The sample points of the 100:0 and 75:25 treatments were relatively close on the first principal component, while those of the 0:100 treatment were more scattered, suggesting that different treatments had varying effects on rice biomass. The arrows (loadings) in the plot showed the degree and direction of the influence of different biomass on the principal components. The root dry weight, shoot dry weight, and total dry weight all exhibited a moderate positive influence on the PC1 (0.4< |loadings|< 0.6). The root dry weight showed a strong positive influence on the PC2 (|loadings| > 0.8), while the shoot dry weight exerted a moderate negative influence on the PC2 (0.4< |loadings|< 0.6).

### 3.4 Cluster analysis

**Figure 4:**
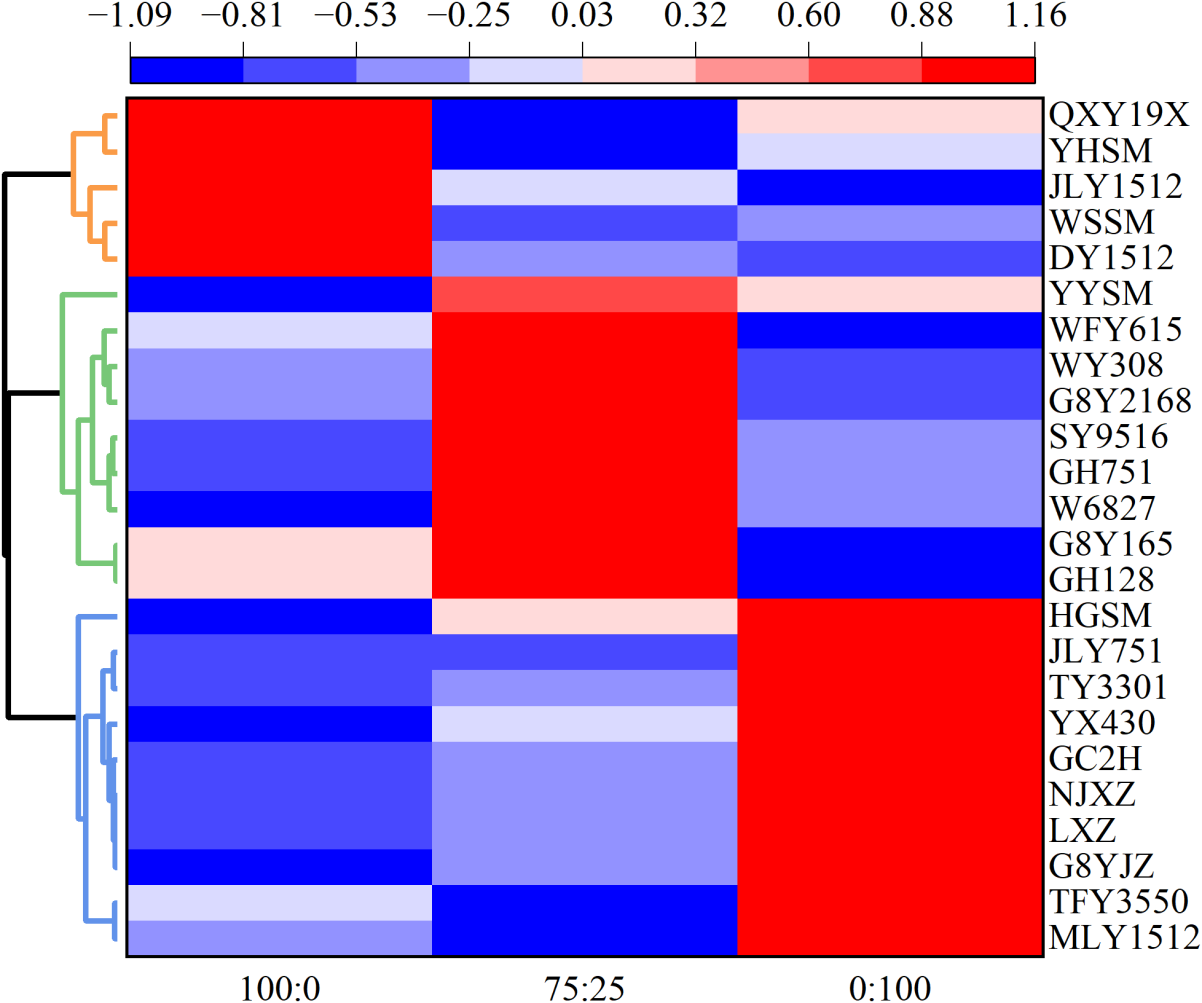
Cluster analysis. The average linkage method and Euclidean distance were used for hierarchical clustering. The cultivar details are provided in Section 2.1. 100:0, 100% ammonium treatment; 75:25, 75% ammonium and 25% nitrate mixed treatment; 0:100, 100% nitrate treatment.

After standardization, the composite scores calculated from the principal component analysis were subjected to cluster analysis, resulting in a cluster heatmap (**Figure 4**). The heatmap categorized the 24 rice cultivars into three types based on their standardized comprehensive scores. Among them, five cultivars, including QXY19X, YHSM, JLY1512, WSSM, and DY1512, presented the highest similarity under the 100:0 treatment but lower similarity under the 75:25 and 0:100 treatments, classifying them as the ammonium-preferring type (APT). Nine cultivars, including YYSM, WFY615, WY308, G8Y2168, SY9516, GH751, W68278, G8Y165, and GH128, exhibited the highest similarity under the 75:25 treatment but lower similarity under the 100:0 and 0:100 treatments, leading to their classification as the ammonium- and nitrate-preferring type (ANPT). Ten cultivars, including HGSM, JLY751, TY3301, YX430, GC2H, NJXZ, LXZ, G8YJZ, TFY3550, and MLY1512, showed the highest similarity under the 0:100 treatment, with lower similarity under the 75:25 and 100:0 treatments, thus they were classified as the nitrate-preferring type (NPT).

### 3.5 Statistical analysis of morphological indicators in rice cultivar types

The three types of rice cultivars shown in **Figure 4** were analyzed for morphological indicators (**Table 2**). ANOVA indicated that only the maximum root length and shoot length were not significantly different (*p* > 0.05) among the types. Additionally, the root dry weight and total dry weight did not significantly differ (*p* > 0.05) across the nitrogen treatments. However, significant interactions between types and nitrogen treatments were detected for biomass (*p* < 0.01). With the same type, the maximum root length and shoot length increased under the treatment with nitrate, whereas the SPAD value decreased under the 0:100 treatment. Specifically, the biomass was the highest in the 100:0 treatment of the APT, in the 75:25 treatment of the ANPT, and in the 0:100 treatment of the NPT, with significant differences observed (*p* < 0.05). Comparing the different types, the biomass of the ANPT was significantly greater than that of the APT (*p* < 0.01).

**Table 2:** Influence of nitrogen treatments on morphological indicators of rice cultivar types.

Types	Nitrogen treatments	MRL (cm)	SL (cm)	SPAD (relative unit)	RDW (mg·plant^–1^)	SDW (mg·plant^–1^)	TDW (mg·plant^–1^)
APT	100:0	14.36 ± 0.70 c	21.38 ± 0.50 a	33.24 ± 0.26 a	3.74 ± 0.14 a	13.17 ± 0.37 a	16.91 ± 0.49 a
	75:25	18.18 ± 0.60 b	21.72 ± 0.42 a	33.33 ± 0.30 a	3.32 ± 0.16 a	10.49 ± 0.46 b	13.81 ± 0.61 b
	0:100	22.20 ± 0.52 a	22.07 ± 0.40 a	31.79 ± 0.26 b	3.49 ± 0.19 a	10.02 ± 0.39 b	13.51 ± 0.54 b
	Average	18.25 ± 0.59 A	21.72 ± 0.25 A	32.79 ± 0.19 A	3.52 ± 0.10 B	11.23 ± 0.31 B	14.74 ± 0.39 B
ANPT	100:0	14.25 ± 0.57 c	20.21 ± 0.57 b	33.84 ± 0.39 a	3.52 ± 0.13 b	12.27 ± 0.52 b	15.79 ± 0.64 b
	75:25	18.79 ± 0.57 b	21.60 ± 0.56 ab	34.07 ± 0.29 a	4.23 ± 0.16 a	14.03 ± 0.48 a	18.27 ± 0.63 a
	0:100	22.47 ± 0.53 a	22.46 ± 0.54 a	32.25 ± 0.35 b	3.80 ± 0.13 b	11.53 ± 0.52 b	15.33 ± 0.63 b
	Average	18.50 ± 0.49 A	21.42 ± 0.34 A	33.39 ± 0.22 A	3.85 ± 0.09 A	12.61 ± 0.31 A	16.46 ± 0.39 A
NPT	100:0	13.78 ± 0.61 c	21.39 ± 0.46 b	33.11 ± 0.38 a	3.43 ± 0.10 b	11.86 ± 0.45 b	15.29 ± 0.54 b
	75:25	17.85 ± 0.58 b	22.03 ± 0.40 b	32.67 ± 0.53 a	3.66 ± 0.13 b	12.12 ± 0.44 b	15.78 ± 0.56 b
	0:100	22.44 ± 0.78 a	23.41 ± 0.38 a	31.89 ± 0.43 a	4.39 ± 0.13 a	14.13 ± 0.48 a	18.52 ± 0.60 a
	Average	18.02 ± 0.53 A	22.28 ± 0.25 A	32.56 ± 0.26 A	3.83 ± 0.08 AB	12.70 ± 0.28 A	16.53 ± 0.36 A
Multifactorial analysis of variance							
Types (T)		NS	NS	3.82*	3.95*	6.33**	6.05**
Nitrogen treatments (N)		113.00**	7.50**	10.81**	3.80*	NS	NS
T×N		NS	NS	NS	8.51**	11.49**	11.38**

The values are presented as the mean ± SE. The least significant difference method was used for multiple comparisons. Different letters in the same column indicate significant differences. Lowercase letters indicate comparisons within the same type (α = 0.05); uppercase letters denote comparisons across different types (α = 0.01). MRL, maximum root length; SL, shoot length; SPAD, SPAD value; RDW, root dry weight; SDW, shoot dry weight; TDW, total dry weight; APT, ammonium-preferring type; ANTP, ammonium-nitrate-preferring type; NPT, nitrate-preferring type; 100:0, 100% ammonium treatment; 75:25, 75% ammonium and 25% nitrate mixed treatment; 0:100, 100% nitrate treatment; Average are the average values of three nitrogen treatments; NS, not significant (*p<* 0.05); *p < 0.05; **p < 0.01.

## 4 Discussion

### 4.1 The role of ammonium-nitrate mixed nutrition

Ammonium and nitrate are vital for plant growth, each with its own benefits and drawbacks. Ammonium stimulates auxin and glutamine synthetase synthesis, thereby benefiting plant photosynthesis (Hessini et al., 2019; Meier et al., 2020; Fortunato et al., 2023). However, prolonged use can acidify the soil and cause ammonium toxicity in plants (Hachiya et al., 2021). On the other hand, nitrate acts as a signaling molecule (Wang et al., 2018), promoting cytokinin synthesis and root growth (Gu et al., 2018; Huo et al., 2020). However, a single application of nitrate decreases the SPAD value (Fu H. Y. et al., 2021), which may be related to the higher energy consumption required for assimilating nitrate (Shilpha et al., 2023) as well as rhizosphere alkalinization and iron deficiency (Chen et al., 2018). Therefore, a mixture of these two nutrients might be used to overcome these limitations and synergistically optimize plant growth when combined.

Numerous studies have reported that a proper balance of ANMN can increase rice root development, including increasing the number and length of lateral and adventitious roots (Song et al., 2011), as well as significantly increasing the tiller number and net photosynthetic rate (Duan et al., 2007). This leads to an increase in rice root dry weight, shoot dry weight and nitrogen accumulation (Zhang et al. 2004b), ultimately increasing total biomass (Ma et al., 2022). The beneficial effects of an appropriate ratio of ANMN are attributed to their ability to regulate the intracellular charge and pH balance in plant cells, increase nitrogen storage, and help plants adapt to adverse conditions (Hachiya and Sakakibara, 2017), thereby promoting overall growth and biomass accumulation (Huo et al., 2020). Additionally, this combination enhances the activity of enzymes crucial for plant growth (Jiang et al., 2021), including those involved in chlorophyll synthesis and nitrogen metabolism. For example, Xu et al. (2020) reported that an appropriate proportion of ANMN, with moderate dry–wet alternating irrigation, improved rice root morphology and activated root nitrogen metabolism enzyme activity and soil enzyme activity. These improvements result in better nutrient uptake, which directly contributes to the increased total biomass and yield (Jiang et al., 2021).

The promoting effect of a suitable ratio of ANMN on rice growth clearly far surpassed that of the single nitrogen treatment. Our results indicate that, compared with the 100:0 treatment, the 75:25 treatment significantly increased the maximum root length, shoot length, and biomass of the rice seedlings. Compared with the 0:100 treatment, the 75:25 treatment significantly increased the leaf SPAD value and shoot dry weight (**Table 1**; **Supplementary Table S1**). This finding confirms that a moderate ratio of ANMN can promote rice growth, likely increase leaf photosynthesis, and consequently increase biomass accumulation.

### 4.2 Ammonium and nitrate preferences of the rice cultivars

Many studies have been conducted on the ammonium and nitrate preferences of rice cultivars (Liu et al., 2016; Chen et al., 2019). Zhang et al. (2004b) reported that both *indica* and *japonica* cultivars exhibit a greater affinity for ammonium than for nitrate. In *indica* rice, better growth was observed under the 100:0 treatment than under the 0:100 treatment, whereas *japonica* presented the opposite trend. Sun et al. (2013) reported that ammonium is more effective at promoting the growth of *indica* rice than nitrate. Zhang and Chu (2020) observed that there are differences in the absorption and assimilation capacity for nitrate between *indica* and *japonica* cultivars, with *indica* rice being more sensitive to nitrate. The contradictory findings in these studies may be attributed to variation in ammonium and nitrate preferences among different cultivars within the same subspecies. Ammonium can promote the synthesis of auxin and nitrogen-metabolizing enzymes, enhancing plant growth (Xie et al., 2023). Xiao et al. (2023) demonstrated that under the condition of pH 4.5, nitrate, rather than ammonium, is more effective at promoting the growth of *indica* rice and regulating the rhizosphere microbial community. Our previous research found that specific rice cultivars preferred ANMN due to upregulating the expression of genes related to carbon-nitrogen metabolism in rice, such as nitrogen metabolism enzymes and amino acid synthesis, thereby promoting the absorption and transport of nitrogen, phosphorus, and potassium (Fu et al., 2023; Fan et al., 2025). However, the possible physiological and genetic mechanism on nitrogen form preference among different cultivars still needs further research.

Sun et al. (2013) reported significant differences under the same nitrogen treatment, regardless of whether the same or different rice subspecies were considered. Specifically, they found that *indica* rice outperformed *japonica* rice, hybrid *indica* rice outperformed conventional *indica* rice, and conventional dry *japonica* rice outperformed conventional wet *japonica* rice in terms of photosynthetic characteristics and nitrogen absorption and utilization. This study demonstrated that there are differences in the response to nitrogen treatments across cultivars, regardless of whether they belong to different subspecies or the same subspecies. Our study revealed significant differences in the response of various *indica* cultivars to nitrogen treatments, as evidenced by the variation in the maximum root length, shoot length, SPAD value, and biomass (**Table 1**; **Supplementary Table S1**). However, the nitrogen preferences of conventional rice and hybrid rice show no clear pattern, which may be related to the genetic makeup of the cultivars (Zhang and Chu, 2020). This research lays a theoretical foundation for classifying rice cultivars based on their ammonium and nitrate preferences.

### 4.3 Classification study of *indica* cultivars and the morphological characteristics of their types

Few studies have classified ammonium and nitrate preferences among rice cultivars. Li et al. (2013) evaluated the biomass, yield, and nitrogen uptake of various crops, including rice, tobacco, and tomatoes, which were primarily treated with either ammonium or nitrate. These crops can be divided into four types: those with a preference for ammonium, those with a preference for nitrate, those with an equivalent response to ammonium and nitrate, and those with a preference for ammonium-nitrate mixtures. Among these types, rice is classified as preferring ammonium. Additionally, it has been suggested that supplementation with a certain amount of nitrate during the reproductive growth stage significantly enhances rice growth and increases yield. On the other hand, Xiao et al. (2016) applied a nitrate-only treatment under both high and low nitrogen levels. Based on the biomass and nitrogen content of *indica* and *japonica* cultivars, they classified the cultivars into four types: high nitrogen-high biomass, high nitrogen-low biomass, low nitrogen-high biomass, and low nitrogen-low biomass. While these studies focused on the classification of different crops or nitrate-only treatment in China, there are no reports on the classification of ammonium and nitrate preferences among the distinctive *indica* cultivars in southern China. Therefore, this study conducted a classification study on 24 *indica* cultivars widely cultivated in southern China, dividing the rice cultivars into three types: APT, ANPT and NPT (**Figure 4**; **Table 2**). This research has significant implications for guiding targeted improvements in rice cultivars and efficient nitrogen fertilizer utilization. It is possible to apply corresponding fertilizers according to the type of rice cultivars to optimize nitrogen use efficiency.

Numerous studies have assessed rice cultivars based on their morphological characteristics. Chen et al. (2017) identified tiller number, total root length, surface area, and volume as crucial indicators for evaluating nitrogen-efficient rice cultivars. Liu et al. (2022) reported that nitrogen-efficient rice cultivars generally present greater root dry weight, a greater number of lateral roots, and increased total root length, root surface area, and root volume. These studies have shown that morphological indicators can be used to assess the characteristics of rice cultivars. However, our study revealed that different rice cultivars exhibited distinct trends in the maximum root length, shoot length, and SPAD value across various nitrogen treatments. Specifically, the maximum root length and shoot length increased under the treatment containing nitrate, whereas the SPAD value notably decreased under the 0:100 treatment (**Tables 1**, **2**; **Supplementary Table S1**). These indicators are obviously biased by the presence of nitrate, rendering them unsuitable for use as classification indicators. Cao et al. (2007) found that compared with those in the 100:0 treatment, the root length, biomass and nitrogen uptake in the 50:50 treatment increased significantly, with nitrogen-efficient cultivars showing greater increases than nitrogen-inefficient cultivars. Specifically, the biomass was significantly and positively correlated with the yield and nitrogen uptake (Fu et al., 2024), making it a crucial indicator for assessing the ammonium and nitrate preferences. In our study, we observed that the biomass was greatest for the APT under the 100:0 treatment, for the ANPT under the 75:25 treatment, and for the NPT under the 0:100 treatment (**Table 2**). These findings suggest that different rice cultivar types have their optimal nitrogen nutrition, indicating significant differences in ammonium and nitrate preferences among different cultivars within the same rice subspecies. The methodology of this study can be adjusted according to the soil and climatic conditions of different regions to adapt to the rice cultivar types in various areas. This classification method provides a theoretical basis and technical guidance for breeding rice varieties with different nitrogen preference types. Additionally, we found that the biomass of the ANPT was significantly greater than that of the APT, differing from the results of Li et al. (2013). This discrepancy could be attributed to differences in classification scope. Their study included a wide range of crops with numerous cultivars, resulting in broader classification. Moreover, their evaluation was primarily based on single ammonium or nitrate nutrition treatments. In contrast, our study focused only on rice, specifically *indica* cultivars in South China, and investigated their responses to ammonium and nitrate preferences. Furthermore, we considered three nitrogen nutrition, resulting in a more specific and precise classification. In conclusion, our study demonstrated that variation in the maximum root length, shoot length, and SPAD value of rice cultivars can be attributed to different nitrogen treatments. Differences in biomass among types reflect the ammonium and nitrate preferences of rice cultivars, highlighting the importance of biomass as a key agronomic indicator for assessing the ammonium and nitrate preferences.

## 5 Conclusions

The maximum root length, shoot length, and SPAD value of rice significantly responded to the changes in nitrogen treatments. Rice cultivars can be divided into three types, APT, ANPT, and NPT, with biomass serving as a specific indicator of the ammonium and nitrate preferences of rice. These findings have substantial implications for targeted breeding high-yield and nitrogen-efficient rice cultivars, optimizing nitrogen fertilizer strategies, and improving nitrogen use efficiency in *indica* rice producing regions.

## Data Availability

The original contributions presented in the study are included in the article/**Supplementary Material**. Further inquiries can be directed to the corresponding authors.
