# Supplementary material for: Unveiling nitrogen preferences in indica rice: a classification study of cultivars in South China
Source: Front Plant Sci. 2025 Apr 28;16:1568383. doi: 10.3389/fpls.2025.1568383 (PMC12066440; doi:10.3389/fpls.2025.1568383)
Supplement: Supplementary file 1 [file DataSheet1.pdf]

Supplementary material

Table S1 Effect of nitrogen treatments and cultivars on morphological indicators of rice seedlings.

Cultivars	Nitrogen treatments	MRL (cm)	SL (cm)	SPAD (relative unit)	RDW ($mg·plant^{-1}$)	SDW ($mg·plant^{-1}$)	TDW ($mg·plant^{-1}$)
MLY1512	100:0	9.59±0.29 c	15.26±0.34 c	33.03±0.39 a	2.60±0.08 b	7.89±0.25 b	10.48±0.17 b
	75:25	13.97±0.33 b	18.49±0.57 b	30.93±0.28 b	2.20±0.06 c	7.74±0.15 b	9.94±0.18 b
	0:100	15.98±0.28 a	20.36±0.25 a	31.90±0.32 ab	3.22±0.06 a	9.40±0.23 a	12.62±0.30 a
G8Y165	100:0	12.84±0.17 c	14.57±0.17 c	33.52±0.15 a	2.85±0.04 b	11.39±0.14 a	14.24±0.18 a
	75:25	17.12±0.43 b	18.48±0.39 b	33.85±0.41 a	3.22±0.07 a	11.38±0.14 a	14.60±0.21 a
	0:100	21.88±0.35 a	20.58±0.16 a	31.93±0.34 b	3.11±0.04 a	9.55±0.07 b	12.66±0.06 b
GH128	100:0	14.40±0.44 c	19.2±0.57 ab	37.87±0.27 a	3.34±0.06 a	10.79±0.17 b	14.13±0.22 a
	75:25	18.62±0.21 b	17.73±0.33 b	37.02±0.43 a	3.38±0.05 a	11.52±0.21 a	14.90±0.26 a
	0:100	21.36±0.27 a	19.80±0.35 a	34.67±0.12 b	3.37±0.06 a	9.59±0.24 c	12.96±0.30 b
W6827	100:0	10.38±0.11 c	18.36±0.16 a	34.70±0.21 b	2.59±0.07 c	8.62±0.29 b	11.21±0.35 b
	75:25	15.81±0.17 b	19.22±0.17 a	34.55±0.34 b	3.77±0.09 a	11.64±0.26 a	15.41±0.35 a
	0:100	18.67±0.20 a	19.42±0.41 a	35.77±0.09 a	3.28±0.09 b	8.48±0.24 b	11.75±0.33 b
GH751	100:0	10.09±0.24 c	19.12±0.27 a	33.67±0.15 b	4.26±0.06 b	11.92±0.23 b	16.18±0.29 b
	75:25	15.03±0.36 b	20.71±0.41 a	34.77±0.14 a	4.63±0.04 a	14.08±0.24 a	18.71±0.28 a
	0:100	18.20±0.21 a	19.84±0.38 a	31.22±0.42 c	4.54±0.06 a	11.57±0.12 b	16.11±0.16 b
DY1512	100:0	15.44±0.43 c	19.03±0.18 b	33.83±0.44 a	3.10±0.07 a	12.44±0.22 a	15.54±0.29 b
	75:25	19.63±0.58 b	20.65±0.45 a	34.25±0.49 a	2.32±0.04 b	8.29±0.20 b	10.61±0.25 b
	0:100	22.76±0.21 a	20.62±0.36 a	31.98±0.26 b	2.19±0.07 b	7.80±0.28 b	9.98±0.35 b
G8Y2168	100:0	13.47±0.36 c	20.61±0.44 b	32.68±0.27 a	2.97±0.07 b	11.18±0.22 b	14.15±0.30 b
	75:25	18.12±0.49 b	22.60±0.38 a	31.65±0.54 a	3.58±0.06 a	14.06±0.32 a	17.64±0.38 a
	0:100	22.55±0.28 a	22.36±0.36 a	29.98±0.49 b	3.01±0.04 b	10.71±0.28 b	13.72±0.32 b
G8YJZ	100:0	11.28±0.11 c	20.06±0.51 a	30.07±0.67 a	2.85±0.07 c	9.18±0.23 b	12.04±0.17 c
	75:25	16.32±0.18 b	21.62±0.39 a	26.17±0.34 c	3.08±0.06 b	9.63±0.10 b	12.70±0.11 b
	0:100	19.27±0.19 a	21.74±0.40 a	28.12±0.26 b	3.48±0.05 a	11.39±0.22 a	14.86±0.26 a
YHSM	100:0	9.50±0.23 c	19.86±0.22 a	33.23±0.27 a	3.43±0.03 b	11.31±0.16 a	14.74±0.17 a
	75:25	14.16±0.19 b	19.96±0.44 a	33.32±0.52 a	3.28±0.04 c	9.30±0.08 b	12.57±0.09 b
	0:100	19.68±0.42 a	20.38±0.34 a	32.17±0.47 a	3.76±0.03 a	9.06±0.13 b	12.82±0.16 b
YX430	100:0	10.00±0.10 c	19.87±0.09 a	32.42±0.17 b	3.16±0.04 c	9.59±0.12 b	12.75±0.16 b
	75:25	13.76±0.23 b	18.81±0.45 b	34.00±0.42 a	3.70±0.06 b	9.97±0.30 b	13.67±0.34 b
	0:100	18.54±0.17 a	20.14±0.19 a	34.58±0.14 a	4.24±0.05 a	11.92±0.57 a	16.16±0.62 a
SY9516	100:0	19.13±0.26 c	20.22±0.36 c	35.73±0.52 a	4.41±0.02 c	16.02±0.27 b	20.43±0.29 b
	75:25	24.23±0.52 b	22.34±0.12 b	33.58±0.35 b	5.60±0.07 a	17.23±0.20 a	22.83±0.28 a
	0:100	26.66±0.21 a	23.21±0.19 a	32.78±0.16 b	4.68±0.09 b	16.14±0.05 b	20.82±0.14 b
GC2H	100:0	17.30±0.53 c	21.23±0.21 b	34.00±0.35 a	4.27±0.08 c	14.77±0.05 b	19.04±0.14 c
	75:25	22.56±0.55 b	23.95±0.54 a	34.22±0.14 a	4.59±0.03 b	15.38±0.23 b	19.97±0.22 b
	0:100	29.09±0.25 a	25.21±0.49 a	33.68±0.29 a	5.62±0.06 a	18.70±0.29 a	24.32±0.33 a
LXZ	100:0	14.69±0.40 c	22.24±0.23 b	30.88±0.07 b	3.39±0.05 b	11.40±0.04 b	14.79±0.08 b
	75:25	18.32±0.54 b	22.73±0.42 ab	31.63±0.28 ab	3.65±0.09 b	11.84±0.16 b	15.49±0.14 b
	0:100	23.11±0.21 a	23.61±0.17 a	32.05±0.30 a	4.24±0.09 a	13.69±0.30 a	17.94±0.39 a
WSSM	100:0	14.03±0.17 c	21.61±0.24 ab	34.27±0.41 a	4.17±0.08 a	13.25±0.30 a	17.42±0.38 a
	75:25	18.39±0.30 b	21.16±0.15 b	34.50±0.46 a	3.89±0.11 a	12.06±0.09 b	15.95±0.10 b
	0:100	20.46±0.44 a	22.30±0.28 a	32.40±0.23 b	4.22±0.03 a	11.37±0.01 c	15.59±0.03 b

Table S1 Effect of nitrogen treatments and cultivars on morphological indicators of rice seedlings.

(Continued)

Cultivars	Nitrogen treatments	MRL (cm)	SL (cm)	SPAD (relative unit)	RDW ($mg·plant^{-1}$)	SDW ($mg·plant^{-1}$)	TDW ($mg·plant^{-1}$)
YYSM	100:0	14.22±0.17 c	21.43±0.26 b	33.05±0.43 a	3.20±0.08 b	10.07±0.09 c	13.27±0.12 c
	75:25	17.52±0.25 b	21.41±0.20 b	33.40±0.32 a	3.93±0.05 a	12.72±0.18 a	16.65±0.16 a
	0:100	22.91±0.29 a	23.69±0.43 a	31.82±0.25 b	4.11±0.07 a	10.72±0.16 b	14.83±0.22 b
TFY3550	100:0	19.77±0.41 c	21.85±0.29 b	33.40±0.55 a	3.53±0.08 b	12.34±0.13 b	15.88±0.14 b
	75:25	22.49±0.34 b	23.12±0.14 a	32.47±0.20 a	3.12±0.06 c	11.73±0.30 b	14.85±0.24 c
	0:100	28.94±0.16 a	23.78±0.54 a	32.60±0.12 a	4.28±0.06 a	14.07±0.31 a	18.35±0.37 a
TY3301	100:0	16.79±0.51 c	22.83±0.38 b	32.20±0.15 a	3.71±0.06 c	15.10±0.31 b	18.80±0.25 b
	75:25	20.98±0.37 b	21.95±0.56 b	31.10±0.35 b	4.10±0.03 b	14.38±0.23 b	18.48±0.26 b
	0:100	25.56±0.23 a	25.19±0.41 a	28.77±0.23 c	4.70±0.09 a	16.44±0.30 a	21.15±0.37 a
WFY615	100:0	18.07±0.51 c	22.85±0.48 b	32.48±0.38 a	3.49±0.04 b	13.06±0.11 b	16.55±0.14 b
	75:25	22.60±0.49 b	25.49±0.53 a	33.27±0.38 a	4.69±0.07 a	15.11±0.21 a	19.80±0.28 a
	0:100	25.54±0.35 a	25.18±0.29 a	30.93±0.08 b	3.46±0.07 b	10.64±0.27 c	14.10±0.35 c
QXY19X	100:0	16.09±0.42 c	22.08±0.42 a	32.02±0.51 a	3.46±0.05 b	13.41±0.16 a	16.88±0.20 a
	75:25	18.27±0.29 b	22.76±0.31 a	32.12±0.23 a	3.17±0.07 c	10.01±0.26 b	13.18±0.33 c
	0:100	23.35±0.24 a	23.89±0.49 a	30.27±0.58 a	4.00±0.08 a	10.28±0.20 b	14.28±0.24 b
NJXZ	100:0	13.03±0.15 c	22.44±0.07 a	32.48±0.29 a	3.09±0.01 c	11.66±0.29 b	14.75±0.30 c
	75:25	15.91±0.15 b	21.42±0.30 b	33.80±0.61 a	3.51±0.02 b	12.03±0.01 b	15.53±0.03 b
	0:100	22.32±0.48 a	22.97±0.39 a	29.55±0.29 b	4.08±0.07 a	15.15±0.13 a	19.23±0.19 a
JLY1512	100:0	16.75±0.34 c	24.32±0.31 a	32.83±0.43 a	4.53±0.02 a	15.45±0.24 a	19.98±0.25 a
	75:25	20.44±0.42 b	24.08±0.35 a	32.48±0.36 a	3.97±0.09 b	12.79±0.22 b	16.75±0.30 b
	0:100	24.74±0.48 a	23.16±0.46 a	32.13±0.33 a	3.27±0.07 c	11.61±0.18 c	14.88±0.25 c
JLY751	100:0	10.75±0.12 c	24.49±0.16 b	37.12±0.14 a	4.14±0.05 c	15.17±0.23 a	19.31±0.26 b
	75:25	16.58±0.43 b	25.64±0.14 a	35.90±0.20 b	4.36±0.01 b	14.62±0.22 a	18.98±0.23 b
	0:100	18.91±0.24 a	26.24±0.28 a	32.55±0.45 c	5.35±0.07 a	15.43±0.26 a	20.78±0.33 a
HGSM	100:0	14.57±0.43 c	23.66±0.25 b	35.53±0.30 a	3.60±0.07 c	11.47±0.24 c	15.07±0.18 c
	75:25	17.57±0.33 b	22.62±0.39 c	36.47±0.41 a	4.26±0.06 b	13.91±0.16 b	18.18±0.21 b
	0:100	22.66±0.28 a	24.84±0.17 a	35.05±0.23 a	4.72±0.08 a	15.07±0.24 a	19.79±0.33 a
WY308	100:0	15.62±0.27 c	25.58±0.17 c	30.88±0.15 b	4.51±0.02 b	17.43±0.07 b	21.95±0.05 b
	75:25	20.03±0.36 b	26.44±0.07 b	34.52±0.42 a	5.30±0.08 a	18.55±0.28 a	23.85±0.36 a
	0:100	24.51±0.15 a	28.03±0.29 a	31.17±0.13 b	4.62±0.02 b	16.38±0.01 c	20.99±0.01 c

The values are presented as the mean±SE. For multiple comparisons, the least significant difference method was used. Different letters in the same column and same cultivar indicate significant differences ($p < 0.05$). MRL, maximum root length; SL, shoot length; SPAD, SPAD value; RDW, root dry weight; SDW, shoot dry weight; TDW, total dry weight. The cultivar details are provided in Section 2.1. 100:0, 100% ammonium treatment; 75:25, 75% ammonium and 25% nitrate mixed treatment; 0:100, 100% nitrate treatment.
